# Machine Learning–Guided Adjuvant Treatment of Head and Neck Cancer

**DOI:** 10.1001/jamanetworkopen.2020.25881

**Published:** 2020-11-19

**Authors:** Frederick Matthew Howard, Sara Kochanny, Matthew Koshy, Michael Spiotto, Alexander T. Pearson

**Affiliations:** 1 Section of Hematology/Oncology, Department of Medicine, The University of Chicago, Chicago, Illinois; 2 Department of Radiation and Cellular Oncology, The University of Chicago, Chicago, Illinois; 3 Department of Radiation Oncology, University of Illinois at Chicago; 4 Department of Radiation Oncology, The University of Texas MD Anderson Cancer Center, Houston

## Abstract

**Importance:**

Postoperative chemoradiation is the standard of care for cancers with positive margins or extracapsular extension, but the benefit of chemotherapy is unclear for patients with other intermediate risk features.

**Objective:**

To evaluate whether machine learning models could identify patients with intermediate-risk head and neck squamous cell carcinoma who would benefit from chemoradiation.

**Design, Setting, and Participants:**

This cohort study included patients diagnosed with squamous cell carcinoma of the oral cavity, oropharynx, hypopharynx, or larynx from January 1, 2004, through December 31, 2016. Patients had resected disease and underwent adjuvant radiotherapy. Analysis was performed from October 1, 2019, through September 1, 2020. Patients were selected from the National Cancer Database, a hospital-based registry that captures data from more than 70% of newly diagnosed cancers in the United States. Three machine learning survival models were trained using 80% of the cohort, with the remaining 20% used to assess model performance.

**Exposures:**

Receipt of adjuvant chemoradiation or radiation alone.

**Main Outcomes and Measures:**

Patients who received treatment recommended by machine learning models were compared with those who did not. Overall survival for treatment according to model recommendations was the primary outcome. Secondary outcomes included frequency of recommendation for chemotherapy and chemotherapy benefit in patients recommended for chemoradiation vs radiation alone.

**Results:**

A total of 33 527 patients (24 189 [72%] men; 28 036 [84%] aged ≤70 years) met the inclusion criteria. Median follow-up in the validation data set was 43.2 (interquartile range, 19.8-65.5) months. DeepSurv, neural multitask logistic regression, and survival forest models recommended chemoradiation for 17 589 (52%), 15 917 (47%), and 14 912 patients (44%), respectively. Treatment according to model recommendations was associated with a survival benefit, with a hazard ratio of 0.79 (95% CI, 0.72-0.85; *P* < .001) for DeepSurv, 0.83 (95% CI, 0.77-0.90; *P* < .001) for neural multitask logistic regression, and 0.90 (95% CI, 0.83-0.98; *P* = .01) for random survival forest models. No survival benefit for chemotherapy was seen for patients recommended to receive radiotherapy alone.

**Conclusions and Relevance:**

These findings suggest that machine learning models may identify patients with intermediate risk who could benefit from chemoradiation. These models predicted that approximately half of such patients have no added benefit from chemotherapy.

## Key Points

**Question:**

Can machine learning survival models predict which patients with intermediate-risk head and neck squamous cell carcinoma would benefit from adjuvant chemotherapy?

**Findings:**

In this cohort study of 33 526 patients, treatment according to 3 machine learning models, trained and validated using the National Cancer Database, was associated with a survival benefit. These models recommended chemoradiation in 44% to 52% of the population.

**Meaning:**

These findings suggest that machine learning models have the potential to better select intermediate-risk patients in need of trimodality therapy, and further study is warranted.

## Teaser

This cohort study evaluates whether machine learning models could identify patients with intermediate-risk head and neck squamous cell carcinoma who would benefit from chemoradiation.

## Introduction

The adjuvant treatment of resected head and neck squamous cell carcinoma (HNSCC) is guided by the European Organisation for Research and Treatment of Cancer (EORTC) 22931 and Radiation Therapy Oncology Group (RTOG) 95-01 randomized clinical trials.^1,2^ Both trials randomized patients with at least 1 adverse prognostic factor after definitive surgery to adjuvant radiotherapy (RT) or cisplatin-based chemoradiotherapy (CRT), and both demonstrated a progression-free survival (PFS) benefit with CRT. The specific adverse risk features qualifying patients for each trial were variable, but an exploratory combined analysis^3^ suggested that extracapsular extension (ECE) and positive surgical margins were the most significant prognostic factors, and patients with 1 of these 2 features derived benefit from CRT.

Consequently, current clinical practice guidelines recommend CRT for patients with positive margins or ECE and list both RT and CRT as treatment options for patients with the other intermediate-risk features studied in the EORTC and RTOG trials.^4^ Real-world practice reflects the equipoise in treatment of these patients with intermediate risk: a retrospective analysis of the National Cancer Database (NCDB) found that approximately half of patients with resected stage III to stage IV disease without positive margins or ECE received CRT, and CRT was associated with better overall survival (OS).^5^ In a subgroup analysis, an increasing magnitude of benefit of chemotherapy was seen with increasing number of involved lymph nodes. Several other studies using NCDB data have further clarified the benefit of adjuvant therapy in patients with intermediate risk. In oral tongue cancers without positive margins or ECE, patients with involvement of 2 or more lymph nodes and/or a pathologic tumor stage 3 to 4 demonstrated a survival benefit with adjuvant CRT.^6^ An examination of patients aged 70 years or younger found a survival benefit for adjuvant CRT for patients with stage III or IV disease without positive margins or ECE, although the benefit was not statistically significant after propensity matching.^7^

Analysis of real-world practice suggests that younger patients with multiple risk factors are more likely to receive CRT,^5^ but exact patient and disease characteristics that determine the benefit from treatment intensification is still uncertain. In traditional survival analysis, the group of patients who benefits most from a therapy can be identified through the use of interaction terms or by splitting data into multiple subgroups. For example, the Meta-analysis of Chemotherapy in Head and Neck Cancer^8^ examined the benefit of adding chemotherapy to locoregional therapy to various age groups, finding no significant benefit in the group aged 71 years or older. However, there can be significant heterogeneity within subgroups; a patient aged 71 years with T4N3 disease will certainly have more benefit from chemotherapy than a patient aged 91 years with T2N0 disease. Thus, novel approaches for survival analysis are needed. Machine learning is a rapidly evolving field of data analysis that has the ability to account for the interaction between numerous features without explicitly specifying interaction terms or analyzing multiple subgroups.^9^ As a form of artificial intelligence, machine learning refers to a broad range of algorithms that can iteratively improve their performance, making predictions that can mimic human decisions. Deep learning is a subset of machine learning wherein increasingly complex features are identified within each level of a multilayered model. Both traditional machine learning and deep learning have been increasingly used to solve complex problems in medicine using large clinical data sets.^10^

Several machine learning models have been specifically developed to analyze right censored survival data. An early approach is the random survival forest (RSF), which uses the ensemble prediction of multiple decision trees to estimate a hazard function.^11^ A recently developed Cox proportional hazards deep learning model, DeepSurv, was shown to improve on personalized treatment recommendations for the RSF model.^12^ The multitask logistic regression is a proportional hazards model that allows for hazards to vary with time, and a deep learning extension of the model was developed that outperforms standard linear survival models.^13^ As compared with traditional survival analysis, these 3 models allow for the prediction of a unique hazard ratio (HR) for a specific treatment for each patient based on individual disease characteristics. We planned a study to assess the ability of these machine learning models to predict survival in the adjuvant treatment of patients with intermediate risk factors and to identify which patients benefit from CRT.

## Methods

### Study Design and Data Source

This was a retrospective cohort study examining the ability of deep learning models to predict outcomes in patients with resected HNSCC without positive margins or ECE, undergoing adjuvant RT or CRT. We used abstracted patient data from the NCDB, the largest clinical cancer registry in the world, which includes approximately 70% of all new invasive cancer diagnoses in the United States.^14^ This study was determined to be exempt from review by the University of Illinois at Chicago institutional review board. No informed consent was obtained from patients because our team did not obtain any patient-level data; we used the anonymized NCDB database curated by the American College of Surgeons and American Cancer Society. This study followed the Strengthening the Reporting of Observational Studies in Epidemiology (STROBE) reporting guideline.

### Study Population and Covariates

**Figure 1.**
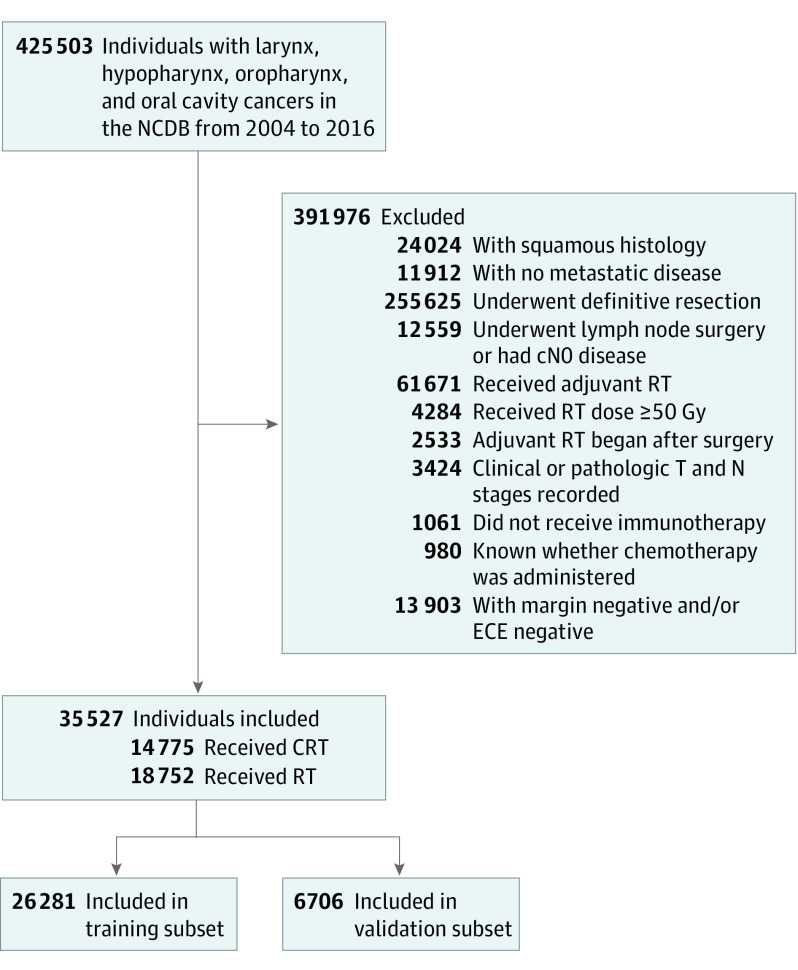
Cohort Selection Criteria CRT indicates chemoradiotherapy; ECE, extracapsular extension; NCDB, National Cancer Database; and RT, radiotherapy.

We included patients diagnosed from January 1, 2004, to December 31, 2016, with squamous cell carcinoma of the oral cavity, oropharynx, hypopharynx, or larynx treated with definitive surgery and adjuvant RT or CRT. We excluded patients with metastatic disease at baseline or those who received immunotherapy. Cohort selection is illustrated in Figure 1.

Feature selection was informed by identifying parameters predictive for survival in a multivariable Cox proportional hazards model. Demographic variables included life expectancy (calculated using the Social Security actuarial life table from 2016^15^), race, sex, treatment at an academic center, year of treatment, and Charlson/Dayo comorbidity index. Disease-specific factors included T stage; N stage; number of lymph nodes involved; involvement of cervical, retropharyngeal, or parapharyngeal lymph nodes; depth of invasion for oral cavity cancers; tumor size; lymphovascular invasion (LVI); grade; HPV status; and primary site/subsite of tumor. Treatment-specific factors included receipt of chemotherapy, use of multiagent chemotherapy, number of lymph nodes dissected, radiation dose, and time from start of adjuvant therapy to completion. Missing categorical values were imputed with the most common category; missing numerical values were imputed with the mean. HPV status was imputed using an extra trees regressor, which predicted the HPV status of known cases with an area under the curve of 0.842.^16^

### Model Development

DeepSurv,^12^ RSF,^11^ and neural network multitask logistic regression (N-MLTR)^13^ models were constructed in Python version 3.7 (Python Software Foundation) using the PySurvival package.^17^ We used an 80:20 split of data for training and validation: 20% of patients were chosen at random to be held out for model validation, whereas all training was done on the remaining 80% of data.^18^ Intrinsic to each model are multiple hyperparameters that may affect predictive accuracy, such as the rate at which the model learns from the data set. To determine the optimal hyperparameters for each model, a random hyperparameter search was performed with 5-fold cross-validation within the training data set, resulting in model parameters (eTable 1 and eTable 2 in the Supplement).

### Statistical Analysis

Statistical analysis was performed in Python version 3.7.5 (Python Software Foundation), and code used for model development and evaluation as well as the trained models generated for this analysis are available online.^19^ The primary outcome was OS benefit associated with treatment according to model recommendations. We considered CRT recommended by a model if predicted survival was longer with CRT than RT. We calculated the HR, median OS, and significance via log-rank test for receipt of treatment in line with model recommendations. The HR was also calculated with inverse probability of treatment weighting (IPTW).^20^ We expected our data set to be imbalanced between those receiving CRT and those receiving RT, with those with higher risk of recurrence likely receiving more intense therapy. IPTW adjusts for this imbalance by weighting cases according to the probability that they received a treatment. We considered multiple secondary outcomes to assess the performance characteristics of these models. Model accuracy was assessed using the concordance index (*C* index),^21^ with confidence intervals assessed using bootstrapping with 1000 iterations^22^ and differences between model *C* indices assessed with analysis of variance. As a comparator for our deep learning models, we used the intermediate risk factors from the EORTC 22931 (T3-4 except T3N0 larynx, N2-3, LVI, deep nodes with oral/oropharynx cancer) or RTOG 95-01 (2 involved nodes) trials as a decision rule. In other words, patients who met the inclusion criteria for these trials would be recommended for chemotherapy, and we calculated the association with OS for such a treatment pattern. Given ongoing attempts to deintensify treatment of HPV-positive cancers as well as the questionable utility of CRT in older patients, the benefit of treatment was assessed in an exploratory analysis of subgroups of patients with HPV-positive cancer and older patients. We also assessed model performance in the subgroups of patients recommended to receive CRT vs RT alone. All comparisons are done at the α = .05 significance level, and all statistical tests were 2-sided when applicable. Because secondary end points were considered exploratory, further adjustment of significance levels for multiple comparisons was not performed. To assess the association of individual features with model accuracy, we calculated the importance of each feature by permutating the data for the feature within the test set. Model accuracy, as indicated by concordance index, is then recalculated with permutated data to determine feature importance.^23^ All data analysis was performed between October 1, 2019, through September 1, 2020.

## Results

A total of 33 527 patients (24 189 [72%] men; 28 036 [84%] aged ≤70 years) met our inclusion criteria, of whom 14 775 (44%) received CRT. Nearly one-fifth of patients (2945 [9%]) received multiagent chemotherapy. Most patients received chemotherapy and radiation concurrently; chemotherapy began more than 30 days prior to radiation in 894 patients (3%). Demographic characteristics of the patients, stratified by receipt of RT or CRT, are summarized in Table 1. Most cancers were of the oral cavity (15 814 [47%]) or oropharynx (11 162 [33%]); nearly one-half had T3 to T4 disease (14 734 [44%]), and approximately two-thirds of patients had lymph node involvement (24 284 [73%]). Distribution of imputed HPV status closely approximated the distribution for cases with known HPV status. Median OS was 97.4 months (95% CI, 94.1-100.7 months) in the RT-only group and 111.2 months (95% CI, 106.8-114.5 months) in the CRT group. Receipt of chemotherapy was significantly associated with male gender, earlier year of diagnosis, lower Charlson/Deyo score, higher T and N stages, greater degree of nodal involvement, and LVI (Table 1). Features with missing data in more than 15% of cases included tumor thickness, measured tumor size, LVI, and HPV status (eTable 3 in the Supplement).

**Table 1.** Patient Demographic, Disease, and Treatment Characteristics

No. (%)	Total population	Received CRT	Received RT	*P* value^a^
Sex				
Men	24 189 (72.1)	11 165 (75.6)	13 024 (69.5)	<.001
Women	9338 (27.9)	3610 (24.4)	5728 (30.5)	
Age, y				
>70	5491 (16.4)	1517 (10.3)	3974 (21.2)	<.001
≤70	28 036 (83.6)	13 258 (89.7)	14 778 (78.8)	
Year of diagnosis				
2004-2008	9697 (28.9)	4461 (30.2)	5236 (27.9)	<.001
2009-2012	10 750 (32.1)	5126 (34.7)	5624 (30.0)	
2013-2016	13 080 (39.0)	5188 (35.1)	7892 (42.1)	
Race				
White	29 261 (87.3)	12 940 (87.6)	16 321 (87)	.10
Black	2801 (8.4)	1229 (8.3)	1572 (8.4)	
Other or unknown	1465 (4.4)	606 (4.1)	859 (4.6)	
Academic center	18 842 (56.2)	7857 (53.2)	10 985 (58.6)	<.001
Charlson/Deyo Score				
0-1	31 752 (94.7)	14 105 (95.5)	17 647 (94.1)	<.001
2-3	1775 (5.3)	670 (4.5)	1105 (5.9)	
Status at last contact				
Alive	21 615 (64.5)	9423 (63.8)	12 192 (65.0)	.06
Dead	11 912 (35.5)	5352 (36.2)	6560 (35.0)	
Primary site				
Oral cavity	15 814 (47.2)	5967 (40.4)	9847 (52.5)	<.001
Buccal mucosa	1082 (3.2)	369 (2.5)	713 (3.8)	
Alveolar ridge	2296 (6.8)	714 (4.8)	1582 (8.4)	
Retromolar trigone	1118 (3.3)	439 (3.0)	679 (3.6)	
Tongue	7207 (21.5)	2822 (19.1)	4385 (23.4)	
Oropharynx	11 162 (33.3)	6086 (41.2)	5076 (27.1)	
Tonsils	7347 (21.9)	4209 (28.5)	3138 (16.7)	
Base of tongue	2997 (8.9)	1488 (10.1)	1509 (8.0)	
Hypopharynx	920 (2.7)	500 (3.4)	420 (2.2)	
Larynx	5631 (16.8)	2222 (15.0)	3409 (18.2)	
Tumor stage				
T1	8544 (25.5)	3859 (26.1)	4685 (25.0)	.02
T2	10 249 (30.6)	4408 (29.8)	5841 (31.1)	
T3	4711 (14.1)	2104 (14.2)	2607 (13.9)	
T4	10 023 (29.9)	4404 (29.8)	5619 (30.0)	
Nodal stage				
N0	11 038 (32.9)	2596 (17.6)	8442 (45.0)	<.001
N1	6674 (19.9)	2578 (17.4)	4096 (21.8)	
N2	15 290 (45.6)	9201 (62.3)	6089 (32.5)	
N3	525 (1.6)	400 (2.7)	125 (0.7)	
Measured tumor size, median (IQR), mm	3.3 (2.0-3.7)	3.3 (2.0-4.0)	3.2 (2.0-3.5)	<.001
Tumor thickness, median (IQR), mm^b^	62.7 (10.0-90.0)	6.46 (11.0-90.0)	6.18 (10.0-90.0)	.01
Differentiation				
Well differentiated	3309 (9.9)	1086 (7.4)	2223 (11.9)	<.001
Moderately differentiated	17 645 (52.6)	7461 (50.5)	10 184 (54.3)	
Poorly differentiated or anaplastic	10 055 (30.0)	4929 (33.4)	5126 (27.3)	
LVI	4654 (13.9)	2449 (16.6)	2205 (11.8)	<.001
Lymph nodes positive				
0	9143 (27.3)	2074 (14.0)	7069 (37.7)	<.001
1	7916 (23.6)	3351 (22.7)	4565 (24.3)	
2-4	13 184 (39.3)	6870 (46.5)	6314 (33.7)	
5-9	2499 (7.5)	1849 (12.5)	650 (3.5)	
≥10	785 (2.3)	631 (4.3)	154 (0.8)	
Lymph node levels involved				
I	6404 (19.1)	3666 (24.8)	2738 (14.6)	<.001
II	14 212 (42.4)	8240 (55.8)	5972 (31.8)	<.001
III	7936 (23.7)	4902 (33.2)	3034 (16.2)	<.001
IV	3047 (9.1)	2086 (14.1)	961 (5.1)	<.001
V	1153 (3.4)	853 (5.8)	300 (1.6)	<.001
Retropharyngeal	265 (0.8)	182 (1.2)	83 (0.4)	<.001
Parapharyngeal	275 (0.8)	165 (1.1)	110 (0.6)	<.001
HPV positivity^c^				
Oropharynx	4038 (77.8)	1994 (77.3)	2044 (78.3)	.77
Nonoropharynx	669 (16.0)	297 (16.9)	372 (15.3)	.13
HPV positivity, imputed^d^				
Oropharynx, %	73.3	74.2	72.4	NA
Nonoropharynx, %	16.7	17.8	16.1	NA
Multiagent chemotherapy	2945 (8.8)	2945 (19.9)	0 (0.0)	
Time from surgery to completion of RT				
>100 d	15 744 (47.0)	7017 (47.5)	8727 (46.5)	.004
≤100 d	17 783 (53.0)	7758 (52.5)	10 025 (53.5)	
RT dose				
50-59 Gy	4734 (14.1)	2535 (17.2)	2199 (11.7)	<.001
60-69 Gy	5848 (17.4)	2230 (15.1)	3618 (19.3)	
≥70 Gy	22 948 (68.4)	9501 (64.3)	13 447 (71.7)	
Adequate lymph node dissection, ie, ≥18+ nodes examined	4731 (14.1)	3044 (20.6)	1687 (9.0)	<.001

Abbreviation: CRT, chemoradiotherapy; HPV, human papillomavirus; IQR, interquartile range; LVI, lymphovascular invasion; NA, not applicable; RT, radiotherapy.

^a^ For categorical values, the *P* value for a χ^2^ test comparing the CRT and RT groups is provided; for numerical values, the *P* value for an unpaired 2-sided *t* test is provided.

^b^ Values listed for the oral cavity subgroup prior to imputation, given that tumor thickness is not available and/or not applicable for other primary tumor sites.

^c^ Percentage listed indicates percentage positive out of all patients from whom HPV status is reported.

^d^ The regressor used to impute HPV status assigns a likelihood from 0 to 1 of HPV positivity for each case. Mean imputed HPV status is listed as a percentage to allow comparison with true HPV rates.

Our models were trained on 26 821 cases, with the remaining 6706 reserved for validation. Results of the hyperparameter search for each model are described in eTable 1 and eTable 2 in the Supplement. With a median follow-up in the validation set of 43.2 (19.8-65.5) months, treatment according to machine learning model recommendations was associated with significantly improved survival for all models (Table 2), with an HR of 0.79 (95% CI, 0.72-0.85; *P* < .001) for DeepSurv, 0.83 (95% CI, 0.77-0.90; *P* < .001) for N-MTLR, and 0.90 (95% CI, 0.83-0.98; *P* = .01) for RSF. No survival benefit was seen with recommending chemotherapy only for patients with intermediate risk who met inclusion criteria for the EORTC 22931 trial (HR, 0.93; 95% CI, 0.86-1.01; *P* = .07) or the RTOG 95-01 trial (HR, 0.96; 95% CI, 0.89-1.05; *P* = .38).

**Table 2.** Model Accuracy and Survival Predictions for Treatment According to Model Recommendations

Model	*C* index	OS, median (IQR), months		HR (95% CI)^a^	*P* value	HR, IPTW (95% CI)^a^	*P* value
		Patients receiving recommended treatment	Patients not receiving recommended treatment				
RTOG 95-01, ≥2 lymph nodes	NA	111.8 (102.1-118.9)	98.1 (91.8-108.9)	0.96 (0.89-1.05)	.38	0.89 (0.81-0.98)	.02
EORTC 22931, T3-4 disease, except T3N0 larynx N2-3, LVI, deep nodes with oral/oropharynx cancer	NA	111.3 (105.4-117.6)	95.3 (86.9-108.2)	0.93 (0.86-1.01)	.07	0.90 (0.82-0.99)	.03
DeepSurv	0.693 (0.675-0.711)	118.1 (111.5-126.5)	90.6 (79.8-98.1)	0.79 (0.72-0.85)	<.001	0.76 (0.69-0.84)	<.001
N-MTLR	0.691 (0.673-0.709)	116.4 (109.7-123.3)	93.5 (85.9-101.1)	0.83 (0.77-0.90)	<.001	0.80 (0.72-0.88)	<.001
RSF	0.695 (0.676-0.713)	111.4 (101.1-120.3)	99.5 (91.7-110.1)	0.90 (0.83-0.98)	.01	0.96 (0.87-1.06)	.41

Abbreviations: EORTC, European Organisation for Research and Treatment of Cancer; HR, hazard ratio; IPTW, inverse probability of treatment weighting; IQR, interquartile range; LVI, lymphovascular invasion; N-MTLR, neural multitask logistic regression; OS, overall survival; RSF, random survival forest; RTOG, Radiation Therapy Oncology Group.

^a^ HRs are given for the patients who received a recommended treatment, compared with those who did not. Results are compared with decision rules derived from the RTOG and EORTC trials, wherein patients were recommended to receive chemoradiation if they met any of the intermediate risk criteria specified by the trials and radiation alone if not.

**Figure 2.**
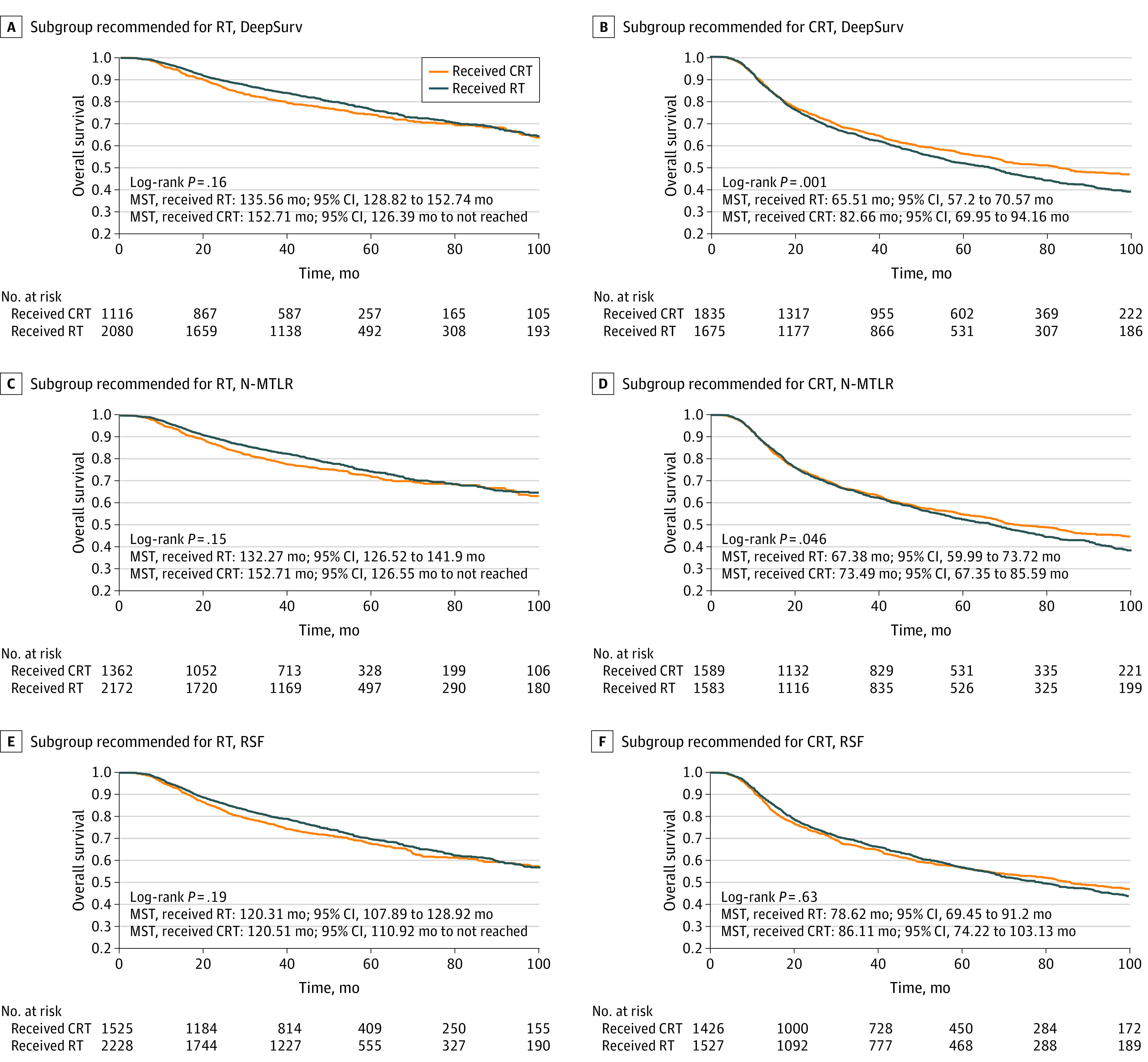
Survival Outcomes for the Subgroup Recommended for Radiotherapy (RT) and chemoradiotherapy (CRT) in the Test Cohort Results illustrated for DeepSurv (A, B), neural network multitask logistic regression (N-MLTR) (C, D), and random survival forest (RSF) (E, F) models. Panels on the left examine the subgroup of patients who were recommended to receive radiation alone by each of the 3 models, with no survival difference (per log-rank test) seen between patients who did or did not receive chemotherapy. A benefit to chemotherapy is seen for patients recommended for CRT by the DeepSurv and N-MTLR models (B, D).

Given the expected differences in patient demographic characteristics among those treated with or without chemotherapy, we repeated these comparisons with IPTW, weighting patients more heavily if they were underrepresented in a given treatment group. Survival benefit remained significant with IPTW for treatment according to the DeepSurv (HR, 0.76; 95% CI, 0.69-0.84; *P* < .001) and N-MTLR (HR, 0.80; 95% CI, 0.72-0.88; *P* < .001) models. Notably, no survival benefit for CRT was seen for patients recommended to receive RT alone in any of the models, suggesting that the models identified a subgroup of patients for whom RT alone is sufficient (Figure 2; eFigure in the Supplement). Within the entire data set, 24 862 patients (74%) met the EORTC 22931 inclusion criteria, and 16 468 (49%) met RTOG 95-01 inclusion criteria. Chemotherapy would be recommended for 17 589 (52%), 15 917 (47%), and 14 912 (44%) of patients in the DeepSurv, N-MLTR, and RSF models, respectively. No significant difference between model accuracy for prognosis was seen among the machine learning models (DeepSurv: *C *index, 0.693; 95% CI, 0.675-0.711; N-MTLR: *C *index, 0.691; 95% CI, 0.673-0.709; RSF: *C *index, 0.695; 95% CI, 0.676-0.713; *P* = .95) (Table 2).

**Figure 3.**
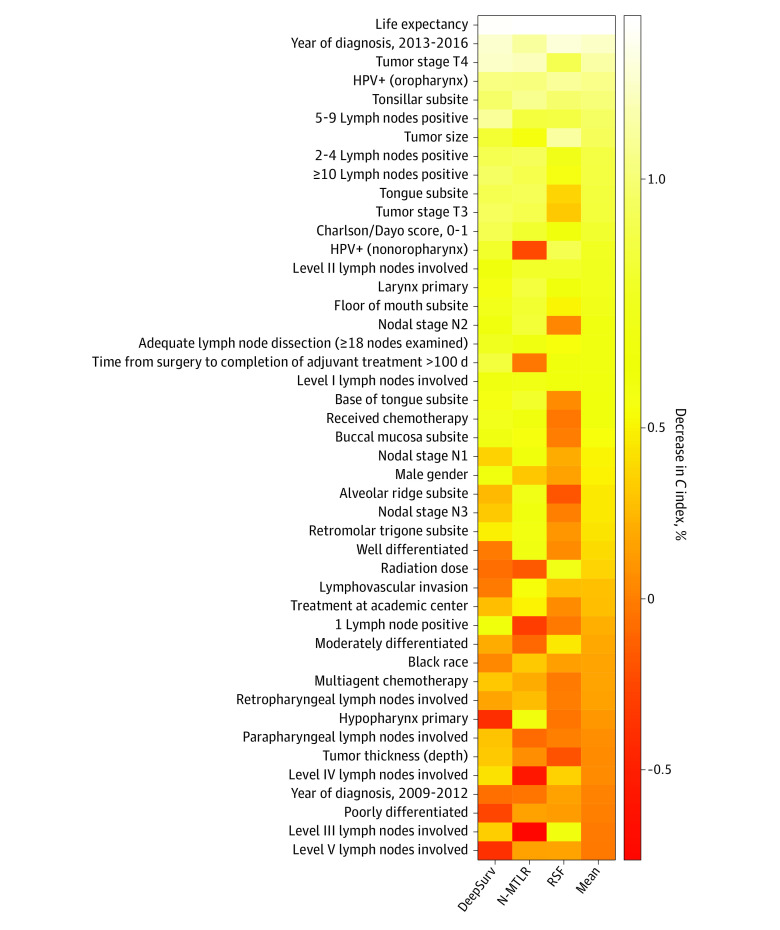
Heatmap for Permutation Feature Importance for DeepSurv, Neural Network Multitask Logistic Regression (N-MLTR), and Random Survival Forest (RSF) Models Values are given as percentage decrease in *C* index, permuted. Higher values indicate greater importance to the predictive accuracy of the respective deep learning model. A log scale is used to highlight the magnitude of each feature’s contribution to overall model accuracy, identifying the most important (>1%), modestly important (>0.1%), less important (>0), and uninformative (<0) features. HPV indicates human papillomavirus.

Assessing variable permutation importance (Figure 3) identified features important to model accuracy for prognosis, with a more than 1% mean reduction in concordance index with permutation of life expectancy, year of diagnosis, T4 tumor stage, HPV positivity, and tonsillar subsite (eTable 4 in the Supplement). In a post hoc analysis, patients in the test cohort were stratified according to HPV status (excluding those for whom HPV status was imputed) and age, and the HR associated with treatment according to model recommendations was measured (eTable 5 in the Supplement). These results suggest that treatment according to DeepSurv recommendations demonstrated a persistent survival benefit in both patients older than 70 years (HR, 0.74; 95% CI, 0.62-0.89; *P* = .002) and those aged 70 years or younger (HR, 0.87; 95% CI, 0.79-0.95, *P* = .003).

## Discussion

Machine learning algorithms have increasingly prominent applications within health care, with applications in head and neck cancer including detection of HPV status from pathology slides,^24^ prospective identification of patients at risk for delay in adjuvant radiotherapy,^25^ and prediction of ECE from imaging.^26^ Deep learning has traditionally excelled at classification tasks, such as identifying objects present in a picture, but the recent development of novel survival models enables new approaches to outcomes data.^12^ Here we demonstrate that adjuvant treatment according to 3 machine learning models can improve patient survival, and all models identified patients who can be safety managed with RT alone. However, only the survival benefit associated with the N-MLTR and DeepSurv models remained significant with IPTW, and only DeepSurv provided robust predictions in both older and younger patients.

Since the results of the EORTC 22931 and RTOG 95-01 trials, there have been numerous attempts to identify subgroups of patients with intermediate risk factors who would benefit from adjuvant CRT. For oral cavity cancer, an observational study^27^ found that patients with 3 intermediate risk factors had a survival benefit from adjuvant CRT. No difference was seen between the RT and CRT groups for patients with only 2 risk factors, but the study was limited because of its observational nature and lack of propensity matching. Indeed, real-world practice would suggest that a significant number of patients with a single intermediate risk factor are not receiving chemotherapy, given that nearly three-quarters of patients in this data set would meet inclusion criteria for EORTC 22931, but only 44% received CRT. All the machine learning models presented here selected a smaller subset of the population than the EORTC 22931 trial for the addition of chemotherapy, and yet they performed at least as well in identifying who would benefit from chemotherapy.

There is particular interest in deescalating patients who have HPV-positive cancers. Several small studies have suggested a lack of association of chemotherapy with outcome in even high-risk patients with HPV-positive cancer.^28,29^ Our models found HPV to be 1 of the most important factors with regard to predictive accuracy. Several ongoing and recently completed randomized clinical trials will help further clarify the management of patients with HPV-positive cancer. Preliminary reports from the phase II ECOG 3311 trial report favorable outcomes for patients with minimal (ie, <1 mm) ECE, fewer than 5 involved lymph nodes, and clear surgical margins treated with postoperative radiation without chemotherapy.^30,31^ The phase III ADEPT trial will examine whether RT alone is adequate for the treatment of patients with HPV with ECE and negative resection margins.^32^ The landscape of HPV-positive cancer is moving toward deintensification, but the lack of randomized data in the intermediate-risk group is a perfect opportunity for machine learning models to identify patients who can forgo chemotherapy.

The benefit of CRT has also been called into question for older patients. Two separate retrospective studies of the NCDB revealed conflicting results regarding even high-risk patients, with 1 analysis finding a survival benefit with CRT and the other failing to find a benefit.^33,34^ However, a clinically relevant trend toward benefit was seen in both studies, suggesting chemotherapy may still have a role in the adjuvant treatment of select older patients. The utility of CRT in older patients with intermediate risk is also unclear, but again, the benefit seen in our DeepSurv model persisted in both young and older patients and could serve as a decision aid in this patient group.

### Limitations

There are several limitations to our current study. The NCDB lacks several important clinical variables, such as smoking history^35^ and perineural invasion,^36^ that could add to our predictive accuracy. Additionally, chemotherapy regimen and dose is not specified in the NCDB beyond the administration of multiagent vs single-agent treatments. The database also has incomplete data for some variables, most prominently HPV status and lymphovascular invasion; undoubtedly a more accurate model could be created if these features were known instead of imputed. Although we controlled for confounders in our data set with IPTW, it is recommended that machine learning models are validated in an independent data set.^37^ Identifying an appropriate external data set is challenging, given that most cases at our center are already represented in the NCDB. Nonetheless, with further study, machine learning models could provide the basis for the prudent use of chemotherapy in patients with intermediate risk.

## Conclusions

In this cohort study, 3 machine learning models predicted which patients with resected HNSCC and intermediate risk would benefit from receiving CRT. While such models are naturally opaque, they excel at identifying novel interactions between data. Future studies will need to confirm the validity of these models, and further analysis with more comprehensive clinical data not captured in the NCDB may result in predictions that are even more accurate. Machine learning has the potential to distill the complex heterogeneity of real-world practice into meaningful recommendations for true precision medicine.

## Notes

Supplement. **eTable 1. **Optimal Hyperparameters, DeepSurv and N-MTLR Models **eTable 2. **Optimal Hyperparameters, Random Survival Forest **eTable 3. **Missing Values for Features in the Final Data Set, Prior to Imputation **eTable 4. **Permutation Feature Importance **eTable 5. **Hazard Ratios for Subgroups **eFigure. **Survival Outcomes for Treatment According to Machine Learning Model Recommendations, Test Cohort

## References

[1] Bernier J, Domenge C, Ozsahin M, ; European Organization for Research and Treatment of Cancer Trial 22931 Postoperative irradiation with or without concomitant chemotherapy for locally advanced head and neck cancer. N Engl J Med. 2004;350(19):1945-1952. doi:10.1056/NEJMoa032641 15128894

[2] Cooper JS, Pajak TF, Forastiere AA, ; Radiation Therapy Oncology Group 9501/Intergroup Postoperative concurrent radiotherapy and chemotherapy for high-risk squamous-cell carcinoma of the head and neck. N Engl J Med. 2004;350(19):1937-1944. doi:10.1056/NEJMoa032646 15128893

[3] Bernier J, Cooper JS, Pajak TF, Defining risk levels in locally advanced head and neck cancers: a comparative analysis of concurrent postoperative radiation plus chemotherapy trials of the EORTC (#22931) and RTOG (# 9501). Head Neck. 2005;27(10):843-850. doi:10.1002/hed.20279 16161069

[4] National Comprehensive Cancer Network (NCCN) Clinical Practice Guidelines Head and neck cancers, version 1.2020 Published February 12, 2020. Accessed March 16, 2020. https://www.nccn.org/professionals/physician_gls/pdf/head-and-neck.pdf

[5] Trifiletti DM, Smith A, Mitra N, Beyond positive margins and extracapsular extension: evaluating the utilization and clinical impact of postoperative chemoradiotherapy in resected locally advanced head and neck cancer. J Clin Oncol. 2017;35(14):1550-1560. doi:10.1200/JCO.2016.68.2336 28475848

[6] Spiotto MT, Jefferson GD, Wenig B, Markiewicz MR, Weichselbaum RR, Koshy M Survival outcomes for postoperative chemoradiation in intermediate-risk oral tongue cancers. Head Neck. 2017;39(12):2537-2548. doi:10.1002/hed.24932 28960621

[7] Osborn VW, Givi B, Rineer J, Patterns of care and outcomes of adjuvant therapy for high-risk head and neck cancer after surgery. Head Neck. 2018;40(6):1254-1262. doi:10.1002/hed.25103 29451961

[8] Pignon J-P, le Maître A, Maillard E, Bourhis J; MACH-NC Collaborative Group Meta-analysis of chemotherapy in head and neck cancer (MACH-NC): an update on 93 randomised trials and 17,346 patients. Radiother Oncol. 2009;92(1):4-14. doi:10.1016/j.radonc.2009.04.014 19446902

[9] Bzdok D, Altman N, Krzywinski M Statistics versus machine learning. Nat Methods. 2018;15(4):233-234. doi:10.1038/nmeth.4642 30100822 PMC6082636

[10] Topol EJ High-performance medicine: the convergence of human and artificial intelligence. Nat Med. 2019;25(1):44-56. doi:10.1038/s41591-018-0300-7 30617339

[11] Ishwaran H, Kogalur UB, Blackstone EH, Lauer MS Random survival forests. Ann Appl Stat. 2008;2(3):841-860. doi:10.1214/08-AOAS169

[12] Katzman JL, Shaham U, Cloninger A, Bates J, Jiang T, Kluger Y DeepSurv: personalized treatment recommender system using a Cox proportional hazards deep neural network. BMC Med Res Methodol. 2018;18(1):24. doi:10.1186/s12874-018-0482-1 29482517 PMC5828433

[13] Fotso S. Deep neural networks for survival analysis based on a multi-task framework. ArXiv. Preprint posted online January 16, 2018. Accessed March 17, 2020. https://arxiv.org/abs/1801.05512

[14] Bilimoria KY, Stewart AK, Winchester DP, Ko CY The National Cancer Data Base: a powerful initiative to improve cancer care in the United States. Ann Surg Oncol. 2008;15(3):683-690. doi:10.1245/s10434-007-9747-3 18183467 PMC2234447

[15] Social Security Administration Actuarial life table. Accessed March 17, 2020. https://www.ssa.gov/oact/STATS/table4c6.html

[16] Geurts P, Ernst D, Wehenkel L. Extremely randomized trees. Mach Learn. 2006;63:3-42. doi:10.1007/s10994-006-6226-1

[17] Fotso S PySurvival: open source package for survival analysis modeling. Accessed March 17, 2020. https://square.github.io/pysurvival/#citation

[18] Raschka S Model evaluation, model selection, and algorithm selection in machine learning. ArXiv. Preprint posted online December 2, 2018. Accessed August 19, 2020. https://arxiv.org/abs/1811.12808

[19] DeepLearningHNSCC. Accessed October 27, 2020. https://github.com/fmhoward/DeepLearningHNSCC

[20] Austin PC, Stuart EA Moving towards best practice when using inverse probability of treatment weighting (IPTW) using the propensity score to estimate causal treatment effects in observational studies. Stat Med. 2015;34(28):3661-3679. doi:10.1002/sim.6607 26238958 PMC4626409

[21] Harrell FE Jr, Lee KL, Califf RM, Pryor DB, Rosati RA Regression modelling strategies for improved prognostic prediction. Stat Med. 1984;3(2):143-152. doi:10.1002/sim.4780030207 6463451

[22] Efron B, Tibshirani RJ An Introduction to the Bootstrap. CRC Press; 1993 Accessed March 21, 2020. https://cds.cern.ch/record/526679/files/0412042312_TOC.pdf

[23] Fisher A, Rudin C, Dominici F. All models are wrong, but many are useful: learning a variable’s importance by studying an entire class of prediction models simultaneously. ArXiv. Preprint posted online December 23, 2019. Accessed March 20, 2020. https://arxiv.org/abs/1801.01489 PMC8323609 34335110

[24] Kather JN, Schulte J, Grabsch HI, Deep learning detects virus presence in cancer histology. bioRxiv. Preprinted posted online July 5, 2019. doi:10.1101/690206

[25] Shew M, New J, Bur AM Machine learning to predict delays in adjuvant radiation following surgery for head and neck cancer. Otolaryngol Head Neck Surg. 2019;160(6):1058-1064. doi:10.1177/0194599818823200 30691352

[26] Kann BH, Aneja S, Loganadane GV, Pretreatment identification of head and neck cancer nodal metastasis and extranodal extension using deep learning neural networks. Sci Rep. 2018;8(1):14036. doi:10.1038/s41598-018-32441-y 30232350 PMC6145900

[27] Chen W-C, Lai C-H, Fang C-C, Identification of high-risk subgroups of patients with oral cavity cancer in need of postoperative adjuvant radiotherapy or chemo-radiotherapy. Medicine (Baltimore). 2016;95(22):e3770. doi:10.1097/MD.0000000000003770 27258508 PMC4900716

[28] Sinha P, Lewis JS Jr, Piccirillo JF, Kallogjeri D, Haughey BH Extracapsular spread and adjuvant therapy in human papillomavirus-related, p16-positive oropharyngeal carcinoma. Cancer. 2012;118(14):3519-3530. doi:10.1002/cncr.26671 22086669

[29] Su W, Liu J, Miles BA, Adjuvant radiation therapy alone for HPV related oropharyngeal cancers with high risk features. PLoS One. 2016;11(12):e0168061. doi:10.1371/journal.pone.0168061 27930732 PMC5145232

[30] Transoral surgery followed by low-dose or standard-dose radiation therapy with or without chemotherapy in treating patients with HPV positive stage III-IVA oropharyngeal cancer. Updated October 26, 2020. Accessed October 27, 2020. https://clinicaltrials.gov/ct2/show/NCT01898494

[31] Ferris RL, Flamand Y, Weinstein GS, Transoral robotic surgical resection followed by randomization to low- or standard-dose IMRT in resectable p16+ locally advanced oropharynx cancer: a trial of the ECOG-ACRIN Cancer Research Group (E3311). J Clin Oncol. 2020;38(15 suppl):6500. doi:10.1200/JCO.2020.38.15_suppl.6500

[32] Post operative adjuvant therapy de-intensification trial for human papillomavirus-related, p16+ oropharynx cancer. Updated November 13, 2019. Accessed March 21, 2020. https://clinicaltrials.gov/ct2/show/NCT01687413

[33] Giacalone NJ, Qureshi MM, Mak KS, Adjuvant chemoradiation does not improve survival in elderly patients with high-risk resected head and neck cancer. Laryngoscope. 2018;128(4):831-840. doi:10.1002/lary.26798 28833217

[34] Woody NM, Ward MC, Koyfman SA, Adjuvant chemoradiation after surgical resection in elderly patients with high-risk squamous cell carcinoma of the head and neck: a National Cancer Database analysis. Int J Radiat Oncol Biol Phys. 2017;98(4):784-792. doi:10.1016/j.ijrobp.2017.03.019 28602410

[35] Osazuwa-Peters N, Adjei Boakye E, Chen BY, Tobo BB, Varvares MA Association between head and neck squamous cell carcinoma survival, smoking at diagnosis, and marital status. JAMA Otolaryngol Head Neck Surg. 2018;144(1):43-50. doi:10.1001/jamaoto.2017.1880 29121146 PMC5833596

[36] Frunza A, Slavescu D, Lascar I Perineural invasion in head and neck cancers - a review. J Med Life. 2014;7(2):121-123. 25408713 PMC4197494

[37] Luo W, Phung D, Tran T, Guidelines for developing and reporting machine learning predictive models in biomedical research: a multidisciplinary view. J Med Internet Res. 2016;18(12):e323. doi:10.2196/jmir.5870 27986644 PMC5238707

