# Supplementary material for: Machine Learning–Guided Adjuvant Treatment of Head and Neck Cancer
Source: JAMA Netw Open. 2020 Nov 19;3(11):e2025881. doi: 10.1001/jamanetworkopen.2020.25881 (PMC7677764; doi:10.1001/jamanetworkopen.2020.25881)
Supplement: Supplement. — eTable 1. Optimal Hyperparameters, DeepSurv and N-MTLR Models eTable 2. Optimal Hyperparameters, Random Survival Forest eTable 3. Missing Values for Features in the Final Data Set, Prior to Imputation eTable 4. Permutation Feature Importance eTable 5. Hazard Ratios for Subgroups eFigure. Survival Outcomes for Treatment According to Machine Learning Model Recommendations, Test Cohort [file jamanetwopen-e2025881-s001.pdf]

Supplemental Online Content

Howard FM, Kochanny S, Koshy M, Spiotto M, Pearson AT. Machine learning–guided adjuvant treatment of head and neck cancer. *JAMA Netw Open*. 2020;3(11):e2025881. doi:10.1001/jamanetworkopen.2020.25881

eTable 1. Optimal Hyperparameters, DeepSurv and N-MTLR Models

eTable 2. Optimal Hyperparameters, Random Survival Forest

eTable 3. Missing Values for Features in the Final Data Set, Prior to Imputation

eTable 4. Permutation Feature Importance

eTable 5. Hazard Ratios for Subgroups

eFigure. Survival Outcomes for Treatment According to Machine Learning Model Recommendations, Test Cohort

This supplemental material has been provided by the authors to give readers additional information about their work.

© 2020 Howard FM et al. *JAMA Network Open.*

eTable 1. Optimal Hyperparameters, DeepSurv and N-MTLR Models

Parameter	DeepSurv Model	N-MTLR Model
**Neural Network Structure**<br><br>**(Layer: activation; nodes)**	1: LeCun Tanh; 64<br><br>2: Sinc; 128<br><br>3: Log Sigmoid; 32<br><br>4: Inverse Square Root; 128<br><br>5: Swish; 16	1: Sinc; 32<br><br>2: Sigmoid; 32<br><br>3: Log Log; 8<br><br>4: Arc Tan; 8
**Learning Rate**	0.00001	0.0001
**Number of Epochs**	6000	4000
**Dropout**	0.1	0.1
**L2 Regularization**	0.0001	0.001
**Batch Normalization**	False	False
**L2 Smoothing Regularization**		0.001
**Bins**		100

© 2020 Howard FM et al. *JAMA Network Open.*

eTable 2. Optimal Hyperparameters, Random Survival Forest

Parameter	Random Survival Forest Model
**Trees**	80
**Max Features**	0.1
**Max Depth**	40
**Min Node Size**	80
**Sample Size Percent**	60%
**Importance Mode**	Permutation

© 2020 Howard FM et al. *JAMA Network Open.*

eTable 3. Missing Values for Features in the Final Data Set, Prior to Imputation

	**Number Missing (% Total)**
**Gender**	0 (0)
**Age**	0 (0)
**Year of Diagnosis**	0 (0)
**Ethnicity**	279 (0.8)
**Academic Center**	1,199 (3.6)
**Charlson/Deyo Score**	0 (0)
**Status At Last Contact**	0 (0)
**Primary Site**	0 (0)
**Tumor stage**	0 (0)
**Nodal Stage**	0 (0)
**Measured Tumor Size**	5,027 (15.0)
**Tumor Thickness (n, % of oral cavity cancers)[a]**	9,670 (61.1)
**Differentiation**	2,518 (7.5)
**LVI**	15,969 (47.6)
**Lymph Nodes Positive**	4,465 (13.3)
**Lymph Node Levels Involved**	
I, II, III	2,211 (6.6)
IV, V, Retropharyngeal	2,269 (6.8)
Parapharyngeal	2,227 (6.6)
**HPV Positivity[b]**	
Oropharynx	5,972 (53.5)

© 2020 Howard FM et al. *JAMA Network Open.*

Non-oropharynx	18,182 (81.3)
**Multiagent Chemotherapy**	1,581 (0)
**Time from Surgery to Completion of RT**	1,508 (4.7)
**RT Dose**	0 (0)
**Adequate Lymph Node Dissection (18+ Nodes Examined)**	79 (0.2)

[a]For tumor thickness, percent missing is calculated as a percentage of oral cavity cancers

[b]For HPV positivity, percent missing values are calculated as a percentage of total oropharynx cancers, and as a percentage of total non-oropharynx cancers.

© 2020 Howard FM et al. *JAMA Network Open.*

eTable 4. Permutation Feature Importance

Given as % decrease in concordance index with permutation. A high reduction in concordance index with variable permutation indicates that feature is important in prediction of prognosis.

Feature	DeepSurv	N-MTLR	RSF	Average
**Life Expectancy**	4.0922	4.4706	4.5369	4.3666
**Year of Diagnosis 2013 - 2016**	2.4761	1.5635	2.8574	2.299
**Tumor Stage T4**	2.3638	2.0986	0.6507	1.7044
**HPV+ (Oropharynx)**	1.157	1.0594	1.4945	1.237
**Tonsillar Subsite**	0.8495	1.2884	0.921	1.0196
**5-9 Lymph Nodes Positive**	1.4671	0.5078	0.5448	0.8399
**Tumor Size**	0.4326	0.1226	1.644	0.7331
**2-4 Lymph Nodes Positive**	0.656	0.729	0.2707	0.5519
**10+ Lymph Nodes Positive**	0.8419	0.6268	0.126	0.5316
**Tongue Subsite**	0.6829	0.7121	0.0724	0.4892
**Tumor Stage T3**	0.7585	0.6258	0.0643	0.4828
**Charlson/Dayo Score 0-1**	0.6489	0.4133	0.1649	0.409
**HPV+ (Non-oropharynx)**	0.4005	-0.048	0.6712	0.3414
**Level II Lymph Nodes Involved**	0.1787	0.3874	0.3788	0.315
**Larynx primary**	0.1357	0.5185	0.1998	0.2847
**Floor of Mouth Subsite**	0.2739	0.4239	0.1008	0.2662
**Nodal Stage N2**	0.2095	0.4524	0.00498	0.2223
**Adequate Lymph Node Dissection (18+ Nodes Examined)**	0.3122	0.227	0.12	0.2197

© 2020 Howard FM et al. *JAMA Network Open.*

**Time from Surgery to Completion of Adjuvant Treatment > 100 Days**	0.4894	-0.00636	0.1667	0.2166
**Level I Lymph Nodes Involved**	0.2214	0.2486	0.1676	0.2125
**Base of Tongue Subsite**	0.1352	0.3989	0.0103	0.1815
**Received Chemo**	0.2981	0.2099	-0.00742	0.1669
**Buccal Mucosa Subsite**	0.2326	0.1229	-0.00064	0.1183
**Nodal Stage N1**	0.071	0.1966	0.039	0.1022
**Male Gender**	0.2082	0.0606	0.029	0.0993
**Alveolar Ridge Subsite**	0.0516	0.2616	-0.039	0.0915
**Nodal Stage N3**	0.0645	0.2072	0.00155	0.0911
**Retromolar Trigone Subsite**	0.0963	0.1469	0.0208	0.088
**Well Differentiated**	-0.00415	0.2316	0.0115	0.0797
**Radiation Dose**	-0.015	-0.032	0.2644	0.0723
**Lymphovascular Invasion**	-0.0024	0.1139	0.0554	0.0556
**Treatment at Academic Center**	0.0538	0.0991	0.0106	0.0545
**1 Lymph Node Positive**	0.1959	-0.061	-0.00494	0.0434
**Moderately Differentiated**	0.0406	-0.021	0.0912	0.0368
**Black Race**	0.00727	0.0638	0.0278	0.0329
**Multiagent Chemotherapy**	0.0628	0.0387	-0.00352	0.0327
**Retropharyngeal Lymph Nodes Involved**	0.0337	0.0527	-0.00041	0.0287
**Hypopharynx primary**	-0.08	0.1568	-0.00887	0.0228
**Parapharyngeal Lymph Nodes Involved**	0.0587	-0.019	0	0.0133
**Tumor Thickness (Depth)**	0.0645	0.0146	-0.04	0.0129

© 2020 Howard FM et al. *JAMA Network Open.*

**Level IV Lymph Nodes Involved**	0.0858	-0.122	0.0695	0.0112
**Year of Diagnosis 2009 - 2012**	-0.0127	-0.00885	0.0287	0.00237
**Poorly Differentiated**	-0.054	0.0304	0.0249	0.000514
**Level III Lymph Nodes Involved**	0.0672	-0.23	0.1524	-0.00361
**Level V Lymph Nodes Involved**	-0.077	0.0299	0.0322	-0.0049

© 2020 Howard FM et al. *JAMA Network Open.*

eTable 5. Hazard Ratios for Subgroups

Hazard ratios are calculated for survival of patients receiving treatment recommended by a model compared to those who did not.

HR (95% CI)	HPV+ Oropharynx	HPV-	Age > 70	Age ≤ 70
**DeepSurv**	0.95 (0.53 – 1.69)	0.79 (0.63– 1.00)	0.74 (0.62 – 0.89)	0.87 (0.79– 0.95)
**N-MLTR**	0.67 (0.66 – 1.04)	0.83 (0.66 – 1.04)	0.86 (0.71 – 1.03)	0.89 (0.81 – 0.98)
**RSF**	0.46 (0.25 – 0.84)	0.80 (0.64 – 1.01)	0.89 (0.74 – 1.06)	0.93 (0.84 – 1.02)

© 2020 Howard FM et al. *JAMA Network Open.*

eFigure. Survival Outcomes for Treatment According to Machine Learning Model Recommendations, Test Cohort

Top – DeepSurv Model, Middle – N-MLTR Model, Bottom – RSF Model.

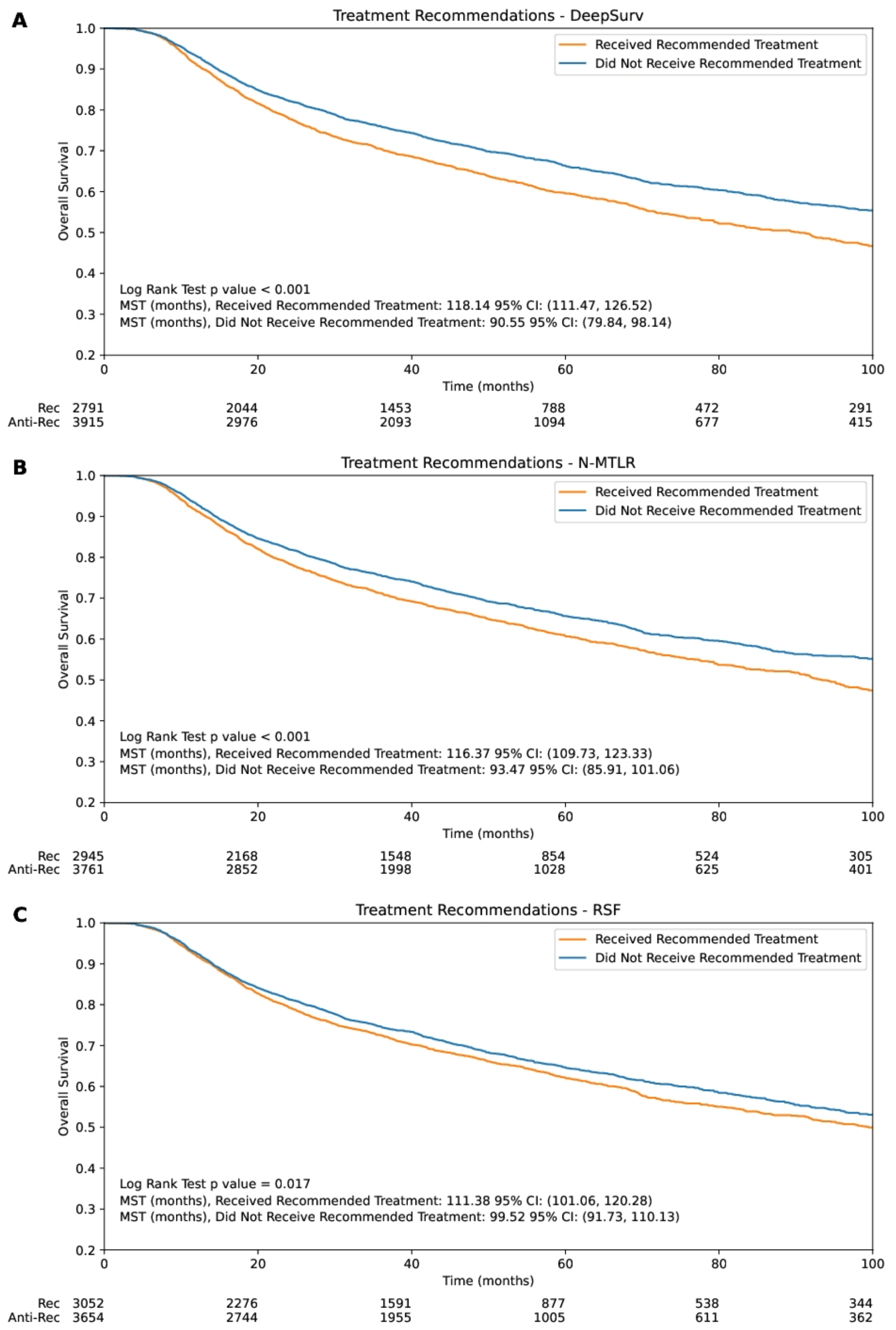

© 2020 Howard FM et al. *JAMA Network Open.*
